# CANNABIS USE TRENDS AND INTENTIONS AMONG OLDER PEOPLE WHO INJECT DRUGS IN LOS ANGELES, CA, AND DENVER, CO, 2021–2022

**DOI:** 10.1093/geroni/igad104.0566

**Published:** 2023-12-21

**Authors:** Ricky Bluthenthal, Jonathan Cohen

**Affiliations:** Keck School of Medicine, University of Southern California, Los Angeles, California, United States; University of Southern California, Los Angeles, California, United States

## Abstract

One potential benefit of drug legalization is that it might lead to substitution of legal products for criminalized substances. This is particularly true in the case of legal cannabis use by the subpopulation of people who inject drugs (PWID) and thus face substantial risk of physical harm, infectious diseases transmission, and even death especially in criminalized environments. Should substitution occur among older PWID, where other comorbid conditions are more common, the benefits could be even greater. To describe substance use patterns among older people who inject drugs and to examine if older PWID are more likely to use cannabis as a substitute for other substances. We analyze data from quantitative, health surveys collected from 429 community-recruited PWID during 2021/22. Descriptive statistics were used to characterize the drug use patterns among older (50 years of age or more) PWID and multivariate regression to examine if older participants were more likely to substitute cannabis for any other substance.

Results: Among the 429 participants, 110 (or 26%) were 50 years of age or older. Older participants reported being significantly less likely than younger participants to use of methamphetamine, fentanyl and cannabis. In terms of cannabis substitution, older participants were significantly more likely to report using cannabis to reduce alcohol use. Risk and benefits of cannabis use vary among subpopulations. Older PWID might benefit from improved access to cannabis when trying to reduce alcohol use. Given the negative consequences of alcohol use, cannabis substitution might have additional benefits for this subpopulation.

